# Assessment Model of Ecoenvironmental Vulnerability Based on Improved Entropy Weight Method

**DOI:** 10.1155/2014/797814

**Published:** 2014-07-15

**Authors:** Xianqi Zhang, Chenbo Wang, Enkuan Li, Cundong Xu

**Affiliations:** ^1^College of Water Resources, North China University of Water Resources and Electric Power, Zhengzhou 450045, China; ^2^Yellow River Institute of Hydraulic Research, Zhengzhou 450003, China

## Abstract

Assessment of ecoenvironmental vulnerability plays an important role in the guidance of regional planning, the construction and protection of ecological environment, which requires comprehensive consideration on regional resources, environment, ecology, society and other factors. Based on the driving mechanism and evolution characteristics of ecoenvironmental vulnerability in cold and arid regions of China, a novel evaluation index system on ecoenvironmental vulnerability is proposed in this paper. For the disadvantages of conventional entropy weight method, an improved entropy weight assessment model on ecoenvironmental vulnerability is developed and applied to evaluate the ecoenvironmental vulnerability in western Jilin Province of China. The assessing results indicate that the model is suitable for ecoenvironmental vulnerability assessment, and it shows more reasonable evaluation criterion, more distinct insights and satisfactory results combined with the practical conditions. The model can provide a new method for regional ecoenvironmental vulnerability evaluation.

## 1. Introduction

As a consequence of both natural changes and human activities, ecoenvironmental vulnerability reflects the sensitive reaction and self-recovery ability in a specific time and space scale [1–3]. Comprehensive analysis and objective assessment on regional ecoenvironmental vulnerability can provide theoretic basis for the sustainable utilization and protection of regional resources, which is of great significance to the regional sustainable development and ecoenvironmental protection. The inland areas in northeast china and northwest china belong to the cold and arid regions, characterized by a typical fragile ecological environment. Recently, with the rapid development of regional society and economy, ecological environment has become increasingly vulnerable, which makes it quite sensitive to external changes. This question has attracted much attention from scholars and researchers [4–7]. Research on ecoenvironmental vulnerability started earlier in foreign countries and now covers a broad range of fields, in which vulnerability assessment has gained favorable achievements [8–11]. Using fuzzy decision method, USEPA (US Environmental Protection Agency) has performed assessment on ecoenvironmental vulnerability in the central regions of Atlantic coast [12]. In addition, lots of methods are used to assess the vulnerability of ecological environment in different regions [13–22]. In China, a lot of researches on ecoenvironmental vulnerability assessment have focused on the cold and arid regions, for example, Ran and Li et al. have performed assessment with the applicability of fuzzy decision analysis method and artificial neutral network [23, 24]. Assessments based on comprehensive evaluation method and analytic hierarchy processes were performed for vegetation vulnerability in Subeinao river basin of Inner Mongolia [25]. Landscape ecology was also used to analyze regional ecoenvironmental vulnerability [26]. In present studies on ecoenvironmental vulnerability, fuzzy evaluation method, artificial neural network, principal component analysis, and comprehensive evaluation method are the most frequently-used techniques [27, 28]. Each method exhibits both advantages and disadvantages; specifically, fuzzy evaluation method overemphasizes the roles of extreme values, which caused huge losses of data information; assessment using artificial neural network is not very precise since they require massive amount of data to support; the comprehensive evaluation function in principal component analysis is not very clear when both negative and positive values exist in the load symbols of main-composition factors; while the repeated utilization ratios of information is relatively high among the indexes in comprehensive evaluation method [29, 30].

Entropy is a thermodynamic conception, which is often used to measure the stage of systemic disorder. It is defined as the information content which can be seen as a negative amount of the logarithm of the probability, an actual promotion on the conception of the original thermodynamic entropy. Information entropy reflects the stage of information disorder, which means the smaller the information entropy of ordered information source and the stage of the disordered system, the greater the utility value of information; the larger of the information entropy of ordered information source and the stage of the disordered system, the smaller the utility value of information. Apparently, decision matrix is a carrier of information, which can be evaluated by using information entropy to obtain the stage of order and utility of information system. Therefore, the use of information entropy model to calculate each index essentially means using the utility value of the index information, and the higher the utility value, the more important the evaluation. That model used to calculate each index above makes it more objective to screen important indicator and compression evaluation system on the maximum based on the evaluation results without losing accuracy.

On the basis of information entropy, the extension of evaluation index number field in normalization has been modified in the present work. An improved entropy weight model on regional ecoenvironmental vulnerability assessment is proposed, which can effectively avoid the losses and overlaps in the hundreds of index information, make the assessment more close to the actual significance, and greatly improve the accuracy and efficiency of assessment.

## 2. Construction of Evaluation Index System

**Figure 1:**
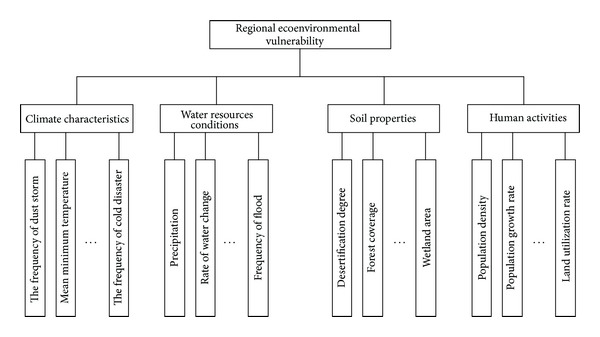
Evaluation index system of ecoenvironmental vulnerability.

Due to a vast territory in China, significant differences exist among different regions in the aspects of natural condition, geography, climate, environment, and underlying surface factors. Apparently, the internal factors and external driving mechanisms in regional ecological environment are different. Therefore, a uniform assessment method regarding ecoenvironmental vulnerability has not yet obtained, partly on account of the difficulties in forming a unified evaluation index system and evaluation method [31]. The influencing factors on ecoenvironmental vulnerability in cold and arid regions are various, while the primary factors especially in northeast and northwest China include the climatic characteristics, water resource conditions, soil properties, and human activities. Accordingly, following the principles of representativeness, science, feasibility, and operability, from the dynamic prospective of external environment and developing variation, the index system of ecoenvironmental vulnerability assessment in cold and arid regions is developed by means of system circulation and hierarchical analysis. This index system aims at assessing regional ecoenvironmental vulnerability, with the climatic characteristics, water resource conditions, soil properties, and human activities adopted as the principles, as shown in Figure 1.

*Climatic Characteristics.* Climatic characteristics play decisive roles in the ecosystem structure and reflect the tolerance and resilience abilities of the ecosystem. The evaluation indexes include the frequency of dust storm, mean minimum temperature, mean maximum temperature, average diurnal temperature difference, cold disaster frequency, aridity, variation rate of aridity index, the frequency of spring drought, the frequency of summer drought, the frequency of cold disaster, and so forth. 

*Water Resources Conditions.* As the source of life, water resources conditions are the basic driving force in the evolution of ecological environment. Changes on water resources conditions will directly affect the ecoenvironmental vulnerability. The evaluation indexes include precipitation, per capita availability of water resources, the quantity of flow per unit area, the pollution rate of water resources, the average index of water shortage, the rate of water change, the frequency of flood, and so forth. 

*Soil Properties.* Soil, the carrier of ecological environment, is also the source of nutrients. The evaluation indexes include the proportion of soil erosion area, desertification degree, diversity of plants and animals, forest coverage, the degree of land desertification, wetland area, and so forth. 

*Human Activities.* The effects of human activities on the carrying capacity of ecological environment are significant. Human activities and ecological environment are both opposite and unified. Human activities are directly related to the degree of ecoenvironmental vulnerability and its developing tendency, with the evaluation indexes including population density, population growth rate, resource consumption per unit GDP (gross domestic product), land salinization degree, grassland degradation rate, land utilization rate, and so forth.

## 3. Improved Entropy Weight Method

According to the administrative division standard, the target region can be divided into multiple small regions, such as provinces, cities, and counties. The different regions are denoted as *X*_*j*_  (*j* = 1,2, 3,…) and the selected indexes are denoted as *X*_1_, *X*_2_,…, *X*_*i*_. The attribute value of the *i*th index in the *j*th region is then denoted as *X*_*ij*_, and the assessment model based on improved entropy weight method can be developed.

During the assessment, different dimensions which exist among different indexes are not comparable with each other. Standardization on the index data is thus required, and the common technique for standardization in entropy method mainly includes range transformation, linear scaling transformation, and vector normalization. Range transformation cannot reflect the correlation among the original indexes objectively, since the differences among the indexes in decision matrix are neglected. Linear scaling transformation is not applicable for a negative index value [32, 33]. By vector normalization, no variation exhibits between the positive and contrary indexes, making the assessment very difficult. *Z*-score (standard score) standardization method is well-adapted for the discrete data, in which the maximum and maximum are not clear or the value exceeds a certain range, and adopted in the present study. The formula in *Z*-score standardization can be expressed as

(1) $$\begin{array}{c} x_{ij}=\frac{\left(X_{ij}-{\overset{-}{X}}_{i}\right)}{S_{i}}, \end{array}$$

where *x*_*ij*_ is the standardized data of the *i*th index in the *j*th region and *X*_*ij*_ is the original data, while *X*_*ij*_ and *S*_*i*_ are the mean value and standard deviation of the *i*th index. To avoid inaccurate calculations on index proportions induced by the cross of positive and negative values among the indexes, the indexes are ensured to be positive with a coordinate transformation method

(2) $$\begin{array}{c} x_{ij}^{\prime }=x_{ij}+A, \end{array}$$

where *x*_*ij*_′ represents the standard value after translation, *x*_*ij*_′ > 0, *A* represents the translational amplitude, *A* > |min⁡⁡(*x*_*ij*_)|. It should be noted that the closer the value *A* to |min⁡⁡(*x*_*ij*_)|, the more significant the assessment result.

The equation to determine the index weight is described as follows:

(3) $$\begin{array}{c} p_{ij}=\frac{x_{ij}^{\prime }}{\sum_{j=1}^{n}x_{ij}^{\prime }}, \end{array}$$

where *p*_*ij*_ is the specific gravity value for each *x*_*ij*_′.

The equation to calculate the index entropy is expressed as follows:

(4) $$\begin{array}{c} e_{i}=-k\sum P_{ij}\ln\left(P_{ij}\right),\;k=\frac{1}{\ln\left(n\right)}, \end{array}$$

where *e*_*i*_ is the *i*th entropy, *k* is a positive value, and *k* = 1/ln⁡⁡(*n*) is selected to ensure that 0 ≤ *e*_*i*_ ≤ 1.

To solve the difference of coefficient among various indicators *g*_*i*_, a smaller entropy coefficient indicates a greater difference among the indicators and a more important index. *g*_*i*_ is calculated by the following equation:

(5) $$\begin{array}{c} g_{i}=1-e_{i}. \end{array}$$

The equation to calculate the weight of indexes is listed as follows:

(6) $$\begin{array}{c} w_{i}=\frac{g_{i}}{\sum_{i=1}^{m}g_{i}}, \end{array}$$

where *w*_*i*_ is the weight of the *i*th indexes.

A comprehensive index *V*_*j*_ to assess the ecoenvironmental vulnerability of a certain *j*th region is calculated as follows:

(7) $$\begin{array}{c} V_{j}=\overset{m}{\underset{i=1}{\sum }}w_{i}P_{ij}\overset{n}{\underset{k=1}{\sum }}w_{k}\left(1-P_{kj}\right), \end{array}$$

where *w*_*i*_ is the weight of the *i*th positive indicator, *P*_*ij*_ is the standard value of the *i*th positive indicator, *w*_*k*_ is the weight of the *k*th contrarian's indicator, and *P*_*ij*_ is the standardized value of the *k*th contrarian's indicator.

## 4. Case Studies

### 4.1. Overview of the Region

Located in the ecotone of agriculture and animal husbandry, the western regions of Jilin province are a typical cold and acid region with fragile ecological environment. The ecological changes in this region are more sensitive to both natural climatic changes and human activities. The target area includes Tiaobei district, Zhenlai county, Tiaonan city, Tongyu county, Da'an city, Ningjiang district, Qianguo county, Qian'an county, Changling county, and Fuyu county in Songyuan City. Annual sunshine duration in this region is approximately 1600~2000 h, annual solar radiation is approximately 5100~5200 MJ*·*m^−2^, the mean precipitation is 400~500 mm, the mean evaporation is 1600~2000 mm, the relative humidity is approximately 60%~65%, and the frostless period is 140~160 d.

The ecological environment problems in this region are mainly characterized by poor weather conditions, frequent natural disasters, three-conversion problems of land (desertification, salinization, and grassland degradation), shortage of water resources, weak water conservancy infrastructure, poor ability to withstand natural disasters, imbalance of industrial structure, and low productions in whole system.

### 4.2. Ecoenvironmental Vulnerability Assessment

According to the construction of the above-described index system and its significance in evaluation system, combined with the data acquisition and data integrity, ten indicators were selected based on climate characteristics, water resources conditions, soil properties, and human activities. The selected indexes in the system include the 1st index: average water shortage; the 2nd index: the change rate of dry index; the 3rd index: the change rate of water; the 4th index: spring drought frequency; the 5th index: summer drought frequency; the 6th index: flood frequency; the 7th index: the degree of grassland degradation; the 8th index: the degree of sand salinization; and the 10th index: the degree of desertification rate.

#### 4.2.1. Construction of Decision Matrix and Standardization

For example, the first value (0.461) of the first row in Table 1 data is the value of the 1st index of Tiao bei. For example, when *i* = 1, *j* = 1, then $x_{11}=(X_{11}-{\overset{-}{X}}_{1})/S_{1}$, the standardized values of the 1st index of Tiao bei can be calculated based on the first column in Table 1 data, with the standardized values according (1) in Table 2. The decision matrix of vulnerability assessment is constructed and displayed in Table 1.

#### 4.2.2. Processing of Decision Matrix

According to the coordinate translation in (7), *A* > |min⁡(*x*_*ij*_)|, and min⁡(*x*_*ij*_) is the minimum value of the data in Table 2. It should be noted that the closer the value *A* to |min⁡(*x*_*ij*_)|, the more significant the assessment result. The minimum value of the data in Table 2 is −2.1209, so assuming that *A* = 2.2, the negative values of coordinate translation parameter in Table 3 are then deleted. According to (2), the proportion values of each index are calculated and shown in Table 4.

#### 4.2.3. Calculation of Index Weight

**Figure 2:**
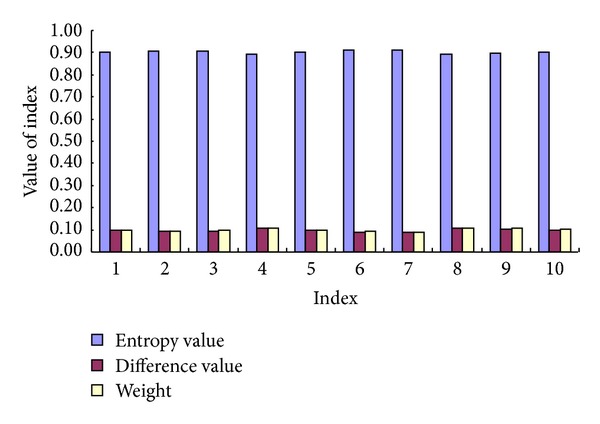
Determination of the index weight.

With the use of the improved entropy method, the index weight is determined according to (4), (5), and (6), as presented in Figure 2.

#### 4.2.4. Integrated Attribute Value Calculation and Evaluation

According to (7), the comprehensive indexes of ecoenvironmental vulnerability in different regions are obtained.

**Figure 3:**
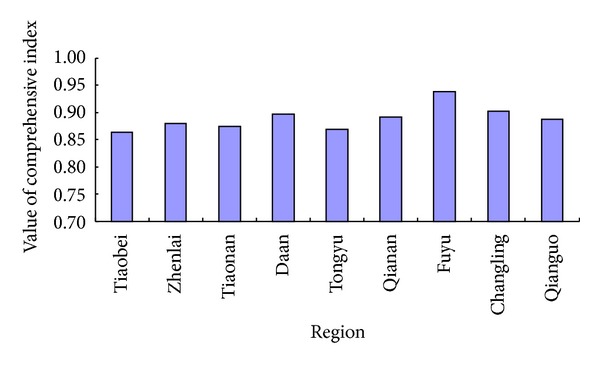
Assessment results.

As shown in Figure 3, the maximum value of partition is 0.9376, appearing in Fuyu country, the minimum value is 0.8636, appearing in Tiaobei district, and the mean value is 0.8889. It can be concluded that the western Jilin province is of a fragile ecological environment. The regions with an ecoenvironmental vulnerability in an aggravating order are Fuyu, Changling, Da'an, Qian'an, Qianguo, Zhenlai, Tiaonan, Tongyu, and Tiaobei.

Matter-element model is put forward by Wen Cai, a Chinese scholar in the 1980s; it is a mathematical method of processing system state transition; it makes “thing,” “features,” and “value” in a continuum considering not only the amount of things, but also the quality of things, with changes of the three elements, the internal structure changes also. This model is widely used in comprehensive evaluation of the environment and ecology. The results by the way of matter element model in an aggravating order is Fuyu, Changling, Da'an, Qian'an, Qianguo, Zhenlai, Tongyu, Tiaonan, and Tiaobei. Conclusively, compared with the results using matter-element model, the assessment results using the method are in good consistence, which indicates that the present method for ecoenvironmental vulnerability assessment is feasible and reasonable.

## 5. Conclusions

Ecoenvironmental characteristics and the driving mechanism should be taken into account in ecoenvironmental vulnerability assessment on the target area, and a reasonable selection of assessment index is of great significance in the construction of a scientific and perfect index system. With regard to the characteristics and evolution mechanisms of the ecological environment in cold and arid regions, the ecoenvironmental vulnerability evaluation index system is established based on the climate characteristics, water resources conditions, soil properties, and human activities. The improved entropy method is then applied to evaluate ecoenvironmental vulnerability combined with a *Z*-score standardization method. With the adoption of improved entropy method, the comprehensive indexes are calculated by the weight of different regions. The experimental results indicate that the model can effectively avoid the equalization index weight and the randomness of subjective weight, and, moreover, the model is suitable for the application of ecoenvironmental vulnerability assessment in the western Jilin province. The regions with an ecoenvironmental vulnerability ranks in an aggravating order are Fuyu, Changling, Da'an, Qian'an, Qianguo, Zhenlai, Tiaonan, Tongyu, and Tiaobei. Conclusively, the results calculated from the present method fit well with the actual situation, suggesting a broad application prospect of the improved entropy method.

## Figures and Tables

**Table 1:** Original data of each region.

Region	1	2	3	4	5	6	7	8	9	10
Tiao bei	0.461	0.246	0.59	90	67.5	20	22.5	5.96	0	75.27
Zhen lai	0.461	0.246	0.4	90.3	71.9	12.5	15.6	3.68	69.86	29.14
Tiao nan	0.432	0.099	1.71	87.1	80.6	9.7	22.6	4.91	62.13	23.46
Da an	0.481	0.099	0	80.6	78.1	9.4	18.8	3.46	45.79	33.32
Tong yu	0.465	0.194	2.96	88.6	83.8	13.9	13.9	4.32	23.55	55.97
Qian an	0.384	0.328	2.32	84.8	67.6	8.8	14.7	2.67	48.3	52.91
Fu yu	0.366	0.099	1.53	77.4	71.9	9.4	15.4	0	26.31	0
Chang ling	0.43	0.076	2.3	68.4	55.3	18.4	15.8	2.46	46.68	77.29
Qian guo	0.383	0.261	1.54	78.4	60.5	15.8	18.3	5.64	40.62	52.51

**Table 2:** Standardized data.

Region	1	2	3	4	5	6	7	8	9	10
Tiao bei	0.8009	0.7247	−0.9611	1.0414	−0.3774	1.7407	1.6229	1.3161	−2.0220	1.2991
Zhen lai	0.8009	0.7247	−1.1655	1.0850	0.1258	−0.1514	−0.6217	0.0013	1.4780	−0.6441
Tiao nan	0.0700	−0.9692	0.2439	0.6193	1.1207	−0.8577	1.6554	0.7106	1.0907	−0.8833
Da an	1.3050	−0.9692	−1.5958	−0.3266	0.8348	−0.9334	0.4193	−0.1256	0.2720	−0.4680
Tong yu	0.9018	0.1255	1.5886	0.8376	1.4866	0.2018	−1.1747	0.3704	−0.8422	0.4861
Qian an	−1.1398	1.6695	0.9001	0.2846	−0.3659	−1.0848	−0.9145	−0.5812	0.3978	0.3572
Fu yu	−1.5935	−0.9692	0.0502	−0.7924	0.1258	−0.9334	−0.6867	−2.1209	−0.7039	−1.8715
Chang ling	0.0196	−1.2342	0.8786	−2.1022	−1.7725	1.3370	−0.5566	−0.7023	0.3166	1.3842
Qian guo	−1.1650	0.8975	0.0610	−0.6468	−1.1779	0.6811	0.2566	1.1316	0.0130	0.3404

**Table 3:** Coordinate translation.

Region	1	2	3	4	5	6	7	8	9	10
Tiao bei	3.0009	2.9247	1.2389	3.2414	1.8226	3.9407	3.8229	3.5161	0.1780	3.4991
Zhen lai	3.0009	2.9247	1.0345	3.2850	2.3258	2.0486	1.5783	2.2013	3.6780	1.5559
Tiao nan	2.2700	1.2308	2.4439	2.8193	3.3207	1.3423	3.8554	2.9106	3.2907	1.3167
Da an	3.5050	1.2308	0.6042	1.8734	3.0348	1.2666	2.6193	2.0744	2.4720	1.7320
Tong yu	3.1018	2.3255	3.7886	3.0376	3.6866	2.4018	1.0253	2.5704	1.3578	2.6861
Qian an	1.0602	3.8695	3.1001	2.4846	1.8341	1.1152	1.2855	1.6188	2.5978	2.5572
Fu yu	0.6065	1.2308	2.2502	1.4076	2.3258	1.2666	1.5133	0.0791	1.4961	0.3285
Chang ling	2.2196	0.9658	3.0786	0.0978	0.4275	3.5370	1.6434	1.4977	2.5166	3.5842
Qian guo	1.0350	3.0975	2.2610	1.5532	1.0221	2.8811	2.4566	3.3316	2.2130	2.5404

**Table 4:** Determination of the index weight values.

Region	1	2	3	4	5	6	7	8	9	10
Tiao bei	0.1516	0.1477	0.0626	0.1637	0.0921	0.1990	0.1931	0.1776	0.0090	0.1767
Zhen lai	0.1516	0.1477	0.0522	0.1659	0.1175	0.1035	0.0797	0.1112	0.1858	0.0786
Tiao nan	0.1146	0.0622	0.1234	0.1424	0.1677	0.0678	0.1947	0.1470	0.1662	0.0665
Da an	0.1770	0.0622	0.0305	0.0946	0.1533	0.0640	0.1323	0.1048	0.1249	0.0875
Tong yu	0.1567	0.1174	0.1913	0.1534	0.1862	0.1213	0.0518	0.1298	0.0686	0.1357
Qian an	0.0535	0.1954	0.1566	0.1255	0.0926	0.0563	0.0649	0.0818	0.1312	0.1292
Fu yu	0.0306	0.0622	0.1136	0.0711	0.1175	0.0640	0.0764	0.0040	0.0756	0.0166
Chang ling	0.1121	0.0488	0.1555	0.0049	0.0216	0.1786	0.0830	0.0756	0.1271	0.1810
Qian guo	0.0523	0.1564	0.1142	0.0784	0.0516	0.1455	0.1241	0.1683	0.1118	0.1283
